# A Behavioral Science-Informed Agentic Workflow for Personalized Nutrition Coaching: Development and Validation Study

**DOI:** 10.2196/75421

**Published:** 2025-09-24

**Authors:** Eric Yang, Tomas Garcia, Hannah G Williams, Bhawesh Kumar, Martin Ramé, Eileen Rivera, Yiran Ma, Jonathan Amar, Caricia Catalani, Yugang Jia

**Affiliations:** 1 Verily Life Sciences, 2999 Olympus Blvd, Ste 1000, Dallas, TX, 75019, United States, 1 650-495-7100

**Keywords:** behavioral science, large language model, LLM, nutritional coaching

## Abstract

**Background:**

Effective management of cardiometabolic conditions requires sustained positive nutrition habits, often hindered by complex and individualized barriers. Direct human management is simply not scalable, and deterministic automated approaches to nutrition coaching may lack the personalization needed to address these diverse challenges.

**Objective:**

We report the development and validation of a novel large language model (LLM)-powered agentic workflow designed to provide personalized nutrition coaching by directly identifying and mitigating patient-specific barriers.

**Methods:**

We used behavioral science principles to create a comprehensive workflow that can map nutrition-related barriers to corresponding evidence-based strategies. First, a specialized LLM agent to intentionally probe for and identify root causes of a patient’s dietary struggles. Subsequently, a separate LLM agent to deliver tailored tactics that were designed to overcome those specific barriers. We conducted a user study with individuals with cardiometabolic conditions (N=16) to inform our workflow design and then validated our approach through an additional user study (n=6). We also conducted a large-scale simulation study, grounding on real patient vignettes and expert-validated metrics, where human experts evaluated the system’s performance across multiple scenarios and domains.

**Results:**

In our user study, the system accurately identified barriers and provided personalized guidance. Five out of 6 participants agreed that the LLM agent helped them recognize obstacles preventing them from being healthier, and all participants strongly agreed that the advice felt personalized to their situation. In our simulation study, experts agreed that the LLM agent accurately identified primary barriers in more than 90% of cases (27 or 28/30). Additionally, experts determined that the workflow delivered personalized and actionable tactics empathetically, with average ratings of 4.17‐4.79 on a 5-point Likert scale.

**Conclusions:**

Our findings demonstrate the potential of this LLM-powered agentic workflow to improve nutrition coaching by providing personalized, scalable, and behaviorally informed interventions.

## Introduction

### Background

The increasing prevalence of cardiometabolic diseases such as diabetes mellitus and hypertension poses a significant global health challenge [1]. Effective management of these conditions hinges on sustained lifestyle modifications, with nutrition playing a central role [2,3]. Digital health interventions, including mobile apps and online platforms, have emerged as readily accessible solutions. These platforms offer nutrition guidance, meal planning tools, and sometimes one-on-one interactions with human experts who aim to promote long-term adherence [4-6]. However, traditional coaching models often face barriers like limited accessibility, high costs, and difficulties in scaling hyper-personalization.

The integration of large language models (LLMs) into digital health nutrition coaching presents an exciting opportunity. LLMs can revolutionize personalized support, optimize intervention effectiveness, and improve access to care. These models can engage in natural language conversations, enabling them to provide dynamic interactions beyond traditional static information delivery. Notably, their conversational ability allows LLMs to clarify user intent, answer nutrition-related questions with contextual nuance, provide tailored recommendations that align with individual preferences and needs, and adapt their approach based on user feedback [7-9]. This personalized conversational approach, combined with the accessibility and scalability of digital platforms, holds immense promise for transforming nutrition coaching and improving the health of individuals around the world. In addition, recent research has showcased LLMs’ potential in gaining user acceptance and delivering accurate domain-specific knowledge, specifically in nutrition applications addressing cardiometabolic conditions [10-13].

Yet, simply delivering nutrition information through general foundation models is unlikely to achieve sustained behavior change. Leveraging the principles of behavioral science is critical for designing sustainable interventions that address the physical, psychological, and social factors influencing dietary choices. Frameworks such as the capability-opportunity-motivation-behavior (COM-B) model are instrumental in understanding the multidimensional factors that contribute to current behavioral patterns [14]. Once the factors preventing behavioral change are better understood, additional frameworks such as the Behavioral Change Wheel, the Behavioral Change Taxonomy (BCT) of 93 hierarchically clustered techniques, and the Easy, Attractive, Social, and Timely (EAST) framework can provide the right approach and tactics on how to promote change [14-16]. These frameworks offer insights into designing interventions that leverage psychological principles to enhance engagement, simplify behavior, make actions rewarding, and trigger positive associations. Combining these complementary frameworks, addressing both the root causes and offering solutions, is crucial to realize LLMs’ potential in delivering sustainable behavioral change.

### Related Work

The application of LLMs specifically to nutrition is a rapidly emerging field. Foundational work has demonstrated their utility for core nutritional analysis tasks, such as generating personalized meal plans based on caloric targets and decomposing compound ingredients for precise analysis [1,2,17,18]. Building on these capabilities, a second stream of research focuses on developing interactive, conversational nutrition assistants. Systems like ChatDiet, for example, use an orchestrator with personal and population data models to deliver highly personalized food recommendations [3,19]. Other work explores multimodality, such as the Purrfessor chatbot which integrates visual meal analysis with conversational advice to enhance user engagement [4,20]. However, while these systems show increasing sophistication in processing nutritional data, their design often overlooks the principles required for sustained behavior change.

Recent research has also explored the potential of LLMs for digital coaching in health behavior change [21-26]. Some literature has reported on interventions that often rely on high-level motivational strategies, utilizing LLMs to deliver advice primarily through broad motivational interviewing techniques or empathetic tones [21,23]. Others have laid groundwork to infuse behavior science principles to the guidance provided by LLMs, incorporating frameworks like COM-B to identify barriers, but their reliance on single conversational turns for barrier classification may limit the depth of their assessments [22]. A separate group of studies has explored LLMs’ ability to leverage external tools effectively to foster user engagement and self-reflection [24,25], or has investigated LLM capabilities to segment users in order to set up different action courses [26].

These separate points of focus in the relevant literature highlight one-by-one the multiple components required for effective full coaching strategies, but several opportunities remain to enhance the integration of behavioral science principles with LLM capabilities, in order to gather individualized user insights and ultimately inform targeted, actionable behavior change tactics. There may be benefits in framing the coaching strategy as a succession of focus points, starting with quantifying barriers from a behavioral science perspective using iterative motivational probing processes, followed by offering specific tactics tailored to individual needs. Overall, there is significant potential for a nuanced integration of behavioral science expertise in the design and development of LLM-powered digital coaching interventions to support more effective and sustainable behavior change.

Our aim in this study was to introduce a novel approach in leveraging LLM-powered nutrition coaching for cardiometabolic condition management by advancing the integration of behavioral science principles and developing a comprehensive, scalable, and expert- and user-validated framework. Our research directly addressed current limitations in the field by creating a multi-agent conversational workflow powered by a deep understanding of nutrition-related barriers and corresponding strategies that directly mitigated the barriers. Through motivational probing, our approach directly identified the root causes of dietary behavior rather than addressing the surface-level symptoms. It fostered a personalized coaching experience, moving beyond high-level motivational techniques and offering targeted tactics supported by behavioral science research. Our approach also enabled a learning system, where barrier-strategy mappings could be tailored to specific individual context, habits, and tactic adoption. We summarize the steps in our study as follows.

### Study Overview

#### User Research and Literature Review for Comprehensive Barrier Identification and Strategy Mapping

We conducted a user research study to identify common nutrition-related barriers experienced by cardiometabolic patients. In addition, we performed a comprehensive literature review of academic research papers and internal reports. We then synthesized these barriers into an overarching set of main barriers encompassing all aspects of nutrition goal achievement. Furthermore, we mapped these barriers to a comprehensive set of strategies and behavioral science tactics, enabling a tailored approach to addressing barrier-specific challenges. This went beyond existing research by providing a more holistic and nuanced understanding of nutrition barriers and their corresponding solutions, paving the way for targeted interventions.

#### Multi-Agent Workflow Design for Personalized Coaching

Building on our comprehensive barrier and strategy mapping and insights from user research, we designed a multi-agent workflow where a specialist LLM agent was tasked with probing and classifying barriers through conversation, while another specialist LLM agent carried out strategies and offered specific tactics. We opted for a multi-agent approach after preceding work (E Yang, MBI, unpublished data, September 2024) showed suboptimal results using a single agent across all the coaching tasks (barrier identification and strategy execution). The present results show that this multi-agent approach improved upon other existing single-agent strategies.

#### Real-World Validation: Collecting Impressions From Cardiometabolic Populations

To ensure practical application and effectiveness, we validated our workflow with participants representative of the cardiometabolic populations we aim to serve. This direct validation provided evidence for the efficacy of our system in addressing real-world nutrition challenges, enhancing the credibility and impact of our research. The real-world validation set our work apart from purely simulation frameworks.

#### Benchmarking and Expert Annotation

Along with behavioral science experts, we used a granular benchmark for evaluating the performance of our LLM agents in various stages, including barrier identification, tactic delivery, and overall conversational attributes. Expert annotations on these metrics provided a rigorous evaluation of our system’s performance.

#### Simulation-Based Evaluation at Scale

We curated real patient vignettes from our user study to generate large-scale simulated conversations of various realistic barrier situations. These data were then evaluated by LLM auto-evaluators, allowing for a scalable and accurate assessment of our system’s performance across a diverse range of scenarios. This simulation-based evaluation approach provided a scalable method for evaluating the generalizability of our LLM-powered coaching system, setting a standard for evaluating similar artificial intelligence (AI)-driven interventions.

## Methods

### Ethical Considerations

Participants provided informed consent and were compensated at the rate of US $100 per hour. Verily Life Sciences research committee performed the ethical review and approved the study plan, declaring the research and resulting dataset exempt. For privacy and confidentiality protection, data were deidentified for analysis and subsequent publication.

### User Research Study

#### Research Objectives

Conducting a user research study was crucial for designing a coaching workflow with the specific needs of individuals with cardiometabolic conditions in mind. By directly involving the target population, we aimed to gain a deeper understanding of realistic nutrition challenges and preferences, ensuring that the system is designed to provide relevant, practical, and effective guidance.

This research study aimed to uncover key user insights necessary for building a patient-centered AI coaching workflow. Specifically, we sought to answer 2 main research questions: (1) What user motivations, characteristics, and challenges must our workflow understand and adapt to in order to be effective? (2) How can an AI coach’s character and conversational patterns inspire trust and engagement while delivering behavioral science strategies?

#### Participants

A total of 16 participants were recruited using third-party services, and following a purposeful sampling strategy, selecting for recent cardiometabolic diagnosis, and otherwise diversity in demographics, US geographic regions, health conditions, attitudes toward health care and technology, and familiarity and comfort with AI. Participants were recruited for sessions across a 12-week period.

#### Procedure

Each participant took part in one-on-one, semi-structured qualitative interviews led by the research team. These interviews focused on participants’ medical histories, the barriers they encountered when working toward their health goals, and their responses to different conversational approaches, as well as their familiarity and general perceptions about AI. Participants shared their experiences and reflected on the specific challenges they faced in managing their health, while also discussing strategies they had found useful. The first step in our research plan, before addressing the second research objective, consisted of participants engaging in interactive exercises where they conversed with 2 types of LLM agents: a supportive agent and an assertive agent (Multimedia Appendix 1, section A). The supportive agent facilitated conversation by treating the user as the expert on their body and experience. This agent encouraged self-reflection and demonstrated high levels of compassion, curiosity, and affirmation. Its tone was plain-spoken, easygoing, and patient, kindly taking direction from the user. In contrast, the assertive agent adopted a directive approach, positioning itself as the resident expert with high energy, authoritative knowledge, and a strategic mindset. This agent aimed to empower the user by being assertive and eager to motivate change. Both agents are powered by Gemini-1.5 Pro, a commercially available model known for its conversational and reasoning capabilities [27]. Their character and tone were guided by specific phrasing and conversational styles in single prompts. From this exercise, we selected the agent type to be carried forward to assemble our agentic workflow. The sessions were conducted on a web-based chat interface, under the observation of user-experience researchers; AI agents were described to participants as “genies” that could work autonomously or in collaboration with clinicians.

To extract output of these study user sessions, analysts used a modified-grounded approach to qualitative analysis, using prior behavior change theories and user experience expertise in the data analysis process. The process began with qualitative coding where analysts labeled sections of data with short words and phrases, capturing what was being communicated. Then, similar codes were grouped together into broader categories that represent recurring patterns. These themes included promoters and detractors of trust and engagement, attitudes toward AI, needs, and expectations. Then, analysts reviewed and refined each theme to ensure fit with matching data and accuracy. Assessing each theme and groups of themes enabled the development of key insights on preferred conversational style. Overall, this approach allowed for a focused exploration of specific aspects of AI desirability and behavior change motivation, while grounding observations on the data itself.

### Behavioral Science Agentic Workflow Design

#### Curation of Barriers and Behavioral Science Strategies

Recognizing that the barriers described by the participants in the user study may not be all-encompassing, we additionally researched the challenges faced by people with cardiometabolic conditions in the literature and marketplace. We analyzed numerous research papers and reports, covering areas like nutrition, medication adherence, exercise, and goal setting. This helped us identify over 100 total barriers individuals experience. Next, we used affinity mapping to organize these barriers, grouping them based on common themes and patterns. By prioritizing the most frequent and impactful challenges, we narrowed our focus to 28 key barriers (Multimedia Appendix 1 section B).

To uncover effective strategies and tactics for addressing these barriers, we conducted a comprehensive review of existing behavioral science frameworks and developed a solutions repository. First, we examined established frameworks, seeking strategies and tactics with proven efficacy in overcoming similar barriers within the health domain. Our review encompassed well-known models like the COM-B model, the BCT Taxonomy, the EAST framework, and other behavioral change models [14-16] (Multimedia Appendix 1 section B). Following this extensive mapping exercise, we curated a final selection of a repository containing over 50 strategies and 100 tactics that are mapped to the 28 barriers. This repository comprehensively linked each identified barrier to a range of potential solutions, offering guidance on optimal implementation to maximize impact. There are popular frameworks and pre-defined sequences for intervention design, which strictly connect the COM-B model to the Behavior Change Wheel to guide the selection of “intervention functions” and “policy domains.” Although we recognize the value of such approaches, we deliberately chose not to explicitly link our workflow to any singular pathways at this stage. We found that the incorporation of other tools, like the 93 Behavioral Change Techniques Taxonomy v1 (BCTv1) and the EAST framework, could be not only suitable but even more appropriate for our population of interest and for our agent capabilities. Ultimately, this decision allowed for greater flexibility and innovation to optimize and personalize the way our agent responded to our population of interest. We could explore the full potential of our future capabilities, ensuring no constraints by any predetermined intervention pathways. A few examples of barriers, strategies, and tactics mapping are shown in Table 1.

**Table 1.** Examples of mapping of barriers, strategies, and tactics.

Barriers	Strategies^a^	Tactics^b^
Decision fatigue: The mental exhaustion from making too many choices can lead to poor, suboptimal future decisions. For example, “As a working parent I have to deal with so many things that I don’t want to think about cooking when I get home.” [28]	Heuristics: Mental heuristics are cognitive shortcuts our brains use to simplify complex situations and make quick decisions [29].	Rules of thumb: Offer the user a set of actionable principles to help them automate tasks. For example, “Always, fill one third of your plate with lean protein.” Default: Encourage the user to pick an option and use it as a default to save time and mental effort. For example, “Let’s set Tuesday as a kale salad day.” [29]
Present bias: It is the user tendency to overvalue immediate rewards over future, larger rewards. For example, “It’s the weekend! Let’s start with dieting next week.” [30]	Future self. Future self is a vivid and emotional connection with a future version of ourselves that affects our intention to engage in a future behavior [31].	Mental rehearsal of successful performance: Encourage the user to practice visualizing themselves successfully performing the desired behavior in realistic scenarios. For example, suggest they vividly imagine eating greens and feeling light to continue with the day [15].

a Strategy: a high-level behavioral science concept aimed to mitigate a barrier.

b Tactic: concrete, tangible, and actionable step to execute on a given strategy.

#### Multi-Agent Workflow Description

**Figure 1.**
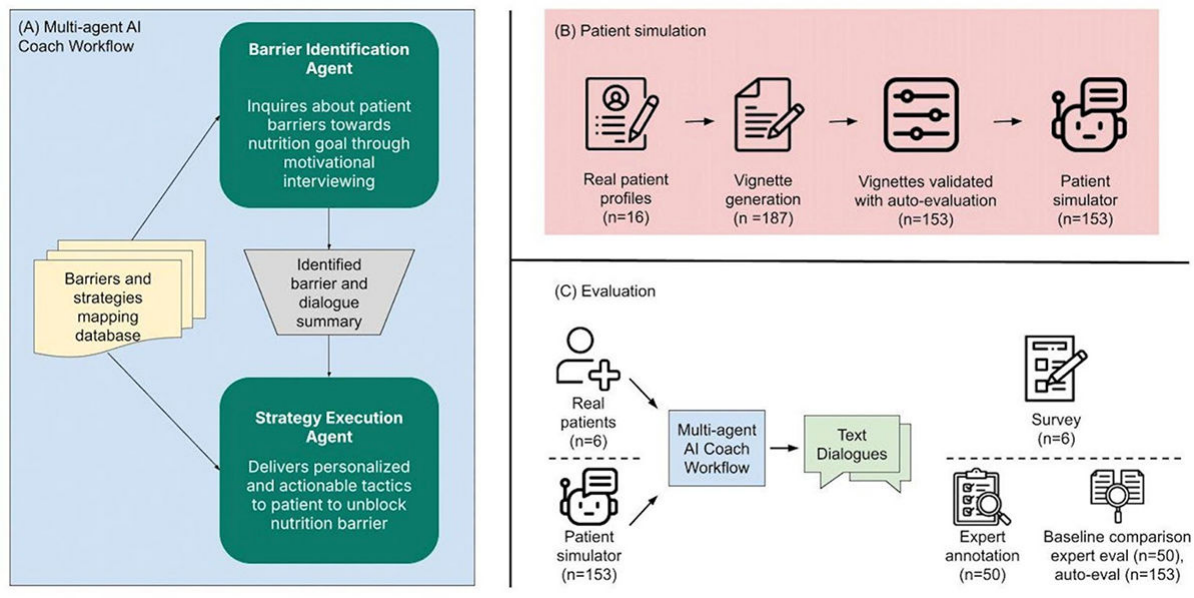
Summary of study steps. (A) The multi-agent artificial intelligence (AI) coach workflow was infused with behavioral science principles to address patients’ barriers toward their nutrition goals. The workflow consisted of 2 core agents, the barrier identification agent and the strategy execution agent. (B) To assess the performance of our workflow at scale, we developed patient simulators that portray validated nutrition vignettes drawn from real cardiometabolic patient profiles. (C) Real cardiometabolic patients and patient simulators interacted with our multi-agent AI coach workflow. The conversation experiences were evaluated via survey, expert annotation of dialogs, and were additionally compared to a baseline large language model (LLM) via auto-evaluation.

This section details the architecture of the multi-agent LLM coaching workflow, aiming to provide tailored nutrition guidance to cardiometabolic patients by identifying and addressing their unique barriers to achieving their nutrition goals. As shown in Figure 1, the system operated through 2 core agents, seamlessly integrated to offer a unified user experience: the barrier identification agent and the strategy execution agent. Both agents were powered by Gemini-1.5 Pro and were prompted to converse in the supportive manner preferred by participants in the user study. We developed the system in Python 3.9 using the Vertex AI API for Gemini. The specific instructions for each agent were defined in system prompts passed during the model’s instantiation, and we used a chat session object to maintain the conversational context.

The barrier identification agent initiated conversations by inquiring about the user’s current nutrition goals and progress. It then employed motivational interviewing techniques to explore the specifics of the user’s nutrition habits, with a focus on identifying the most prominent barrier that may be hindering progress toward their stated goal. The agent was equipped with a predefined taxonomy of 28 barrier concepts, provided within the prompt alongside detailed descriptions and examples. Through iterative dialog, the agent analyzed the user’s responses to classify the identified struggles into one of the barrier concepts. While a user could exhibit multiple barrier concepts, the agent was instructed to prioritize and focus on the most prominent one displayed by the user. By tasking the agent with the identification of a primary barrier (as opposed to relying on users themselves), we circumvented burdening users with triaging technical barrier concepts with which they may be unfamiliar, or sorting through voluminous associated tactic lists that may be overwhelming. Once the agent determined that sufficient information had been gathered for barrier classification, it generated an internal summary of the conversation and the identified barrier concept, which was then relayed to the strategy execution agent. The prompt used by the barrier identification agent can be found in Multimedia Appendix 1, section C.1.

The strategy execution agent received the conversation summary and the identified barrier concept from the barrier identification agent. The agent relied on a structure where each barrier was mapped to a strategy (a high-level behavioral science concept aimed to mitigate a barrier) and, ultimately, to a list of tactics (concrete, tangible, and actionable steps to execute on that strategy) to be carried out by the strategy execution agent. Therefore, each barrier concept eventually mapped to a set of potential tactics, each with examples and an associated execution sequence outlining mandatory and optional tactics, as well as their prioritization. This way, once the agent received the input about the barrier, it retrieved the corresponding tactics and execution sequences from a predefined table. The agent then engaged in further conversation with the user, drawing upon the preceding conversation summary and the prescribed tactics. For the optional tactics, the agent dynamically adapted its offerings based on user responses and perceived acceptability and effectiveness of the previously deployed tactics. The conversation concluded when the agent determined that the user was sufficiently equipped with the necessary tools to overcome their identified barrier and progress toward their nutrition goals. The prompt used by the strategy execution agent can be found in Multimedia Appendix 1, section C.2.

Agent orchestration was defined as when the back-end functionality relied on 2 core distinct agents, the user interacted with the system as a single, continuous AI-powered nutrition coach. This seamless transition between barrier identification and strategy execution was facilitated by the orchestration of information exchange between agents. Crucially, conversation summaries and key outputs, including identified barriers related to nutrition, were explicitly transferred between the agents, ensuring the preservation of all necessary context while excluding irrelevant banter. This modular design allowed each agent to specialize in its respective task, enabling more focused model improvements for both barrier identification and strategy execution. An example of the agent orchestration can be found in Multimedia Appendix 1, section D.2.

#### Evaluation of User Impressions of Workflow

##### Research Objectives

Having implemented the AI coaching workflow grounded on the insights from the initial user research, we conducted further sessions to evaluate users’ impressions of the workflow through direct interactions. This phase aimed to test the real-world applicability and accuracy of the system’s barrier identification and strategy execution capabilities, providing a critical assessment of how well the workflow translates theoretical principles into practical outcomes. Engaging with users also helped us uncover any usability or communication insights that may not have been apparent in agentic workflow and prompt development. Key questions guiding this evaluation were as follows:

##### Effectiveness of Behavioral Science-Informed Workflow

How did users who are managing health concerns respond to our workflow infused with behavior science frameworks? How effectively did our workflow help users identify obstacles that prevent them from being healthier? Were the strategies and tactics offered by our workflow easy for users to put into action? How did our workflow impact users’ motivation and confidence to make positive changes in their health?

##### Building Trust and Engagement Through AI Interactions

Did our AI coach’s character and personality inspire trust and engagement? To what degree did users feel supported by our coaching workflow?

##### Participants

We selected a subset of 6 participants out of the 16 in the user research study described in the prior section, for direct conversational interaction with our LLM coaching workflow in a single session. This scaled-down sample allowed us to conduct the study in a timely fashion. The subset was selected on the basis of diversity with respect to AI comfort levels and stated motivation levels. The participants were primed to think of challenges they might face achieving a nutrition goal they had set, and to channel that while interacting with our coaching workflow. After the experience, the participants were asked to reflect on their interactions through qualitative interviews and to complete a customized survey for this study. Analysts used a modified-grounded approach to qualitative analysis.

### Simulation Study

#### Patient Vignettes

To assess the performance of our multi-agent LLM coaching workflow at a larger scale, we conducted a simulation study with the high-level workflow illustrated in Figure 1. Leveraging patient profiles curated from the user research study that contained patient lifestyle context and medical history, we identified the prominent nutrition barriers for each profile, aligning them with our predefined taxonomy of 28 barrier concepts. This involved a manual process of translating patients’ self-reported nutrition struggles into corresponding barrier concepts. Subsequently, for each identified barrier within each profile, we crafted a detailed patient nutrition vignette paragraph with a separate base Gemini model. These vignettes aimed to vividly depict the specific manifestation of that barrier within the context of the individual’s profile. Providing prewritten nutrition vignettes allowed us to:

#### Focus the Patient Simulator

Our approach enabled the downstream patient simulator to concentrate on portraying the specific barrier and engaging in a meaningful conversation, without the need to generate complex nutrition narratives dynamically.

#### Enhance Control and Consistency

We enabled greater control over the simulated conversations, minimizing the risk of inconsistencies or incoherent narratives that might arise from on-the-fly story generation.

#### Isolate Barrier Identification Impact

##### Overview

Our approach allowed us to isolate and assess the system’s ability to accurately identify and address specific barriers, independent of other confounding factors within a patient’s profile.

In total, we simulated a wide range of scenarios across diverse patient profiles, nutrition goals, and barriers, resulting in 187 total simulated vignettes. We provide a sample vignette generated from a barrier concept for a patient profile in Multimedia Appendix 1, section D.1.

To ensure the quality and relevance of the generated vignettes with respect to the simulated behavioral barrier, we leveraged OpenAI’s GPT-4o (gpt-4o-2024-08-06) model in an LLM-as-judges framework, also known as auto-evaluation [32]. The GPT-4o model was intentionally chosen as the vignettes were generated by a Gemini model. The auto-evaluator received two inputs: the target barrier to be simulated and the generated patient vignette. It then evaluated the vignette across four dimensions: evidence, realism, completeness, and leakage.

##### Evidence (High/Medium/Low)

The extent to which the vignette provided clear indications that the patient’s behavior or thoughts were influenced by the target barrier.

##### Realism (High/Medium/Low)

The plausibility and believability of the depiction of the target barrier, reflecting how it might manifest in a real person’s life.

##### Completeness (High/Medium/Low)

The sufficiency of details provided in the vignette to fully understand the impact of the target barrier on the patient’s ability to achieve their nutrition goals.

##### Leakage (Yes/No)

Whether the vignette directly mentioned the technical term of the target barrier. It was important that vignettes do not explicitly contain the technical terms, which may skew the barrier identification task down the line.

### Conversation Simulation

Having established a robust set of patient vignettes, we proceeded to simulate dialogs between a Gemini-powered patient simulator and our LLM coaching workflow for each of the 153 higher quality vignettes. The patient simulator was provided with two key inputs: the generated vignette from the corresponding patient profile and barrier, and the communication style observed for that patient during the user research study. Notably, the patient simulator’s nutrition barriers were only portrayed via the vignette without explicit mentioning of any technical behavioral science barrier terms. The patient simulator, guided by these inputs, engaged in a conversation with the AI coach. The dialog continued until the AI coach determined, based on its internal logic and the patient’s responses, that the patient had reached a higher level of preparedness to address their nutrition-related challenges. This endpoint represented a complete coaching interaction within the simulation framework. An example of simulated conversations can be found in Multimedia Appendix 1, section D.2.

### Expert Assessment

#### Overview

Evaluating the quality and effectiveness of the simulated coaching conversations was a crucial step in assessing the overall performance of our multi-agent LLM workflow. We leaned on our behavioral science expertise to develop an evaluation rubric encompassing 5 key dimensions: barrier identification accuracy, tactic comprehensiveness, tactic personalization, tactic actionability, and conversation empathy. These dimensions were chosen to reflect the core competencies required for effective digital coaching in the context of motivating dietary behavioral change.

#### Barrier Identification Accuracy

Given the list of 28 nutrition barrier concepts, the AI coach identified the correct patient barrier. Accurate identification of the patient’s primary barrier is paramount for delivering tailored and effective interventions. Rating options: “Yes” or “No.”

#### Tactic Comprehensiveness

The AI coach delivered all the instructed primary tactics to the patient via clear descriptions or relevant examples in an understandable way. Ensuring the delivery of all intended coaching tactics is crucial for maximizing the potential impact of the intervention. Rating options: “Yes” or “No.”

#### Tactic Personalization

For the tactics that were delivered, the AI coach made the tactics personalized to the patient’s unique context. Personalizing coaching tactics to the individual’s unique context and circumstances enhances engagement and promotes behavior change. Rating options: 5-point Likert scale.

#### Tactic Actionability

The AI coach discussed actionable steps to help patients overcome their barriers toward their nutrition goals. Providing clear, actionable steps empowers patients to translate intentions into concrete behaviors. Rating options: 5-point Likert scale.

#### Conversation Empathy

The AI coach provided encouragement and motivation to the patient empathetically. Expressing empathy and providing emotional support fosters a positive and trusting coach-patient relationship. Rating options: 5-point Likert scale.

The corresponding conversations that were simulated for the 50 randomly selected generated vignettes were assessed by human experts with extensive academic training and professional experience in behavioral science. Specifically, two behavioral science experts independently labeled 30 simulated conversations each, with 10 overlapping conversations used to assess inter-rater reliability.

### Comparative Study

To evaluate the advantages of our multi-agent LLM workflow infused with behavioral science principles, we conducted a comparative study against a single base Gemini model that lacked explicit behavioral science knowledge infusion and did not employ a multi-agent approach. The base model was instructed to assist patients in overcoming their nutrition challenges by leveraging its inherent capabilities to apply motivational interviewing and behavioral science tactics without structured guidance. We simulated coaching conversations with the same patient simulator used previously.

Then, a GPT-4o auto-evaluator was employed to determine preference between the 2 conversation sets based on behavioral science criteria. To ensure a fair comparison, we measured the conversation lengths across both conversation sets, ensuring that they contained a comparable number of characters to control for LLM preference for longer contexts. The order in which the conversations were presented to the evaluator was alternated to additionally control for any position bias.

To generate an additional reference data point, the same two behavioral science experts from the Expert Assessment exercise independently and blindly evaluated a randomly shuffled subset of 30 pairs (10 overlapping) of these simulated conversations, namely, conversations from our multi-agent LLM workflow and from the basic Gemini model.

## Results

### User Research Study

We enrolled N=16 participants in this portion of our study (Table 2).

**Table 2.** Characteristics of the study participants (N=16).

Characteristics	Value, n (%)
Sex	
Female	8 (50)
Male	8 (50)
Age (y)	
<45	5 (31.3)
45‐65	9 (56.3)
>65	2 (12.5)
Race/ethnicity	
American Indian/Alaskan Native	1 (6.3)
Black/African American	6 (37.5)
Hispanic/Latino	2 (12.5)
White	7 (43.8)
Recent^a^ cardiometabolic diagnoses	
Hypertension	9 (56.3)
Prediabetes	4 (25)
Type 2 diabetes	10 (62.5)
Hyperlipidemia	2 (12.5)
Obesity	15 (93.8)

a Diagnosis within the last 6 months, n=13; diagnosis more than 6 months ago, n=3.

The user research study revealed crucial insights into the barriers and motivations that should be integrated into the design of the AI coaching workflow. Participants shared detailed accounts of their physical, psychological, and social challenges in achieving their health goals. Common themes that emerged included balancing competing priorities, dealing with physical limitations, and struggling with low self-efficacy. For example, participants frequently expressed feeling overwhelmed by the demands of daily life, which made it difficult to sustain motivation to work on their health over time.

Additionally, participants highlighted the importance of feeling understood and empowered by a coach. A preference emerged for the supportive agent, as most participants felt more comfortable and engaged with its empathetic approach. They noted that the supportive agent fostered trust and provided a space for self-reflection. Although a few participants appreciated the assertive agent’s high-energy style, the majority found it to be less aligned with their desire for a collaborative and self-empowering experience. This feedback was instrumental in refining the AI coaching workflow, which ultimately prioritized the compassionate, user-driven style of the supportive agent to promote long-term engagement and behavior change.

Informed by these findings, we established two guiding principles for the agentic workflow design. First, the workflow must move beyond generic advice to probe and understand the root causes of a user’s barriers, ensuring that subsequent strategies are practical, incremental, and tailored to their personal context. Second, every interaction must be delivered through the supportive, conversational style that participants confirmed was essential for fostering trust and engagement.

### User Impressions of Workflow

#### Effectiveness of Behavioral Science-Informed Workflow

The AI coaching workflow, grounded in behavioral science principles, was found to be effective in addressing barriers users have toward healthier habits. First, the AI coach was successful in helping users identify specific barriers to their health. Five out of 6 participants agreed that the assistant helped them recognize obstacles that prevented them from being healthier. Participants also indicated that their interactions with the AI coach were informative. When asked whether they learned something new about their health habits, 4 out of 6 participants strongly agreed, and the remaining somewhat agreed with the sentiment. As a participant reflected, the agent’s approach of leading with additional questions and building from user experiences enabled “more refined, bite-sized” understanding, enhancing quality of the nutrition motivations and barriers insights collected. Furthermore, participants responded positively to the strategies and tactics provided by the AI coach. All participants strongly agreed that the AI coach’s advice felt personalized to their situation, reflecting its ability to adapt to users’ unique needs. Notably, all participants agreed that the AI coach’s advice was easy to put into action, highlighting its practicality. Participants appreciated the focus on manageable, small changes, with one stating, “Making small changes makes a big difference,” highlighting the perceived effectiveness of the behavior change strategies recommended by the AI coach. The AI coach’s ability to offer novel suggestions tailored to individual preferences was highlighted by participants, such as when one participant appreciated the recommendation to swap quinoa porridge for oatmeal, and another found value in being advised on alternatives to high-sugar protein drinks. The impact of these strategies on user motivation was significant, with all participants agreeing that the AI coach increased their confidence to make positive changes in their health. Each participant also reported feeling more motivated after their conversations due to excitement for having a plan to implement manageable, novel, small changes.

#### Building Engagement Through AI Interactions

Overall, the AI coaching workflow fostered relationship building and engagement through their conversational style and empathetic personality. All participants strongly agreed that they felt supported by the AI coach and were interested in having future conversations about their health, indicating a high level of engagement. One participant noted, “I could sit there and talk to that thing all day.” Furthermore, participants frequently described the AI coach as having a “friendly” and “human-like” demeanor, with one participant mentioning, “It did feel like you’d spoken with somebody, like you had an actual conversation with somebody.” As one participant noted, the AI coach “seemed to be friendly, kind of human-like,” creating a sense of connection and understanding that facilitated deeper discussions about their nutrition habits. The AI coach’s ability to create a supportive environment was evident, with all participants agreeing that they felt comfortable discussing their health issues with them. This sense of comfort was further supported by the finding that the great majority of participants strongly agreed that their conversations were engaging. The AI coach’s empathetic and person-centered communication style not only facilitated open dialog but also encouraged continued user interaction, which is crucial for sustained engagement and positive health outcomes. The full survey results can be found in Multimedia Appendix 1, section E.

### Simulation Study

#### Assessment of the Patient Vignette Auto-Evaluator

To calibrate the auto-evaluator’s performance, we conducted an adversarial analysis. We evaluated the auto-evaluator on a patient vignette paired with a randomly chosen behavioral barrier different from the one simulated in the vignette. This mismatch was designed to assess the auto-evaluator’s sensitivity to inconsistencies between the intended barrier and the generated narrative. We hypothesized that scores for these adversarial cases would be significantly lower, reflecting the lack of alignment between the vignette and the incorrect barrier. The results of this analysis are presented in Table 3.

**Table 3.** Auto-evaluator performance on matched (N=187) and mismatched vignettes (N=187).

	Matched vignette, n (%)			Mismatched vignette, n (%)		
Dimension	High	Medium	Low	High	Medium	Low
Evidence	184 (98.4)	3 (1.6)	0	36 (19.3)	32 (17.1)	119 (63.6)
Realism	187 (100)	0	0	84 (44.9)	94 (50.3)	9 (4.8)
Completeness	156 (83.4)	31 (16.6)	0	19 (10.2)	46 (24.6)	122 (65.2)

Of the 187 simulated vignettes, 153 of them received high marks across evidence, realism, and completeness dimensions by the auto-evaluator. These higher quality vignettes were selected for downstream conversation simulation. As hypothesized, the auto-evaluator assigned significantly lower scores to the mismatched vignettes across all dimensions. This indicates the auto-evaluator’s ability to discern between accurately and inaccurately represented barriers, supporting its validity for assessing vignette quality. In addition, there was no barrier term concept leakage across all cases. Our vignette generation and validation processes provided a robust foundation for our simulation study, ensuring realistic and diverse scenarios for evaluating the effectiveness of our multi-agent LLM coaching workflow.

#### Expert Assessment

The evaluation of the simulated coaching conversations by human experts provided important insights into the performance of our multi-agent LLM workflow. Full results are shown in Table 4. For Barrier Identification Accuracy, the experts agreed that the AI coach accurately identified the primary patient barrier in greater than 90% of cases (in 27/30 cases for 1 reviewer and 28/30 for the other), and the cases with low accuracy score were due to another barrier being deemed more prominent over the one selected by the agent, not to an inability of the agent to identify a barrier. For Tactic Comprehensiveness, the AI coach successfully delivered all instructed primary tactics in 70% of conversations (21/30) labeled by expert 1% and 90% of conversations (27/30) labeled by expert 2. The inter-rater reliability for both dimensions was high, with agreement percentages of 80%, indicating strong consistency between the experts’ assessments. In addition, the agentic workflow received high scores across the remaining dimensions. For Tactic Personalization, the AI coach demonstrated a strong ability to tailor its coaching tactics to the individual’s unique context, with average ratings of 4.38 and 4.79 on a 5-point Likert scale. Tactic Actionability was also rated highly, with average scores of 4.17 and 4.59, reflecting the clarity and feasibility of the steps recommended by the AI coach. Finally, Conversation Empathy received high marks, with average scores of 4.58 and 4.76, indicating that the AI coach was perceived as empathetic and supportive, effectively fostering a positive coaching relationship. While Expert 2 gave slightly higher ratings on average, the absolute difference in ratings for overlapping cases was minimal, reinforcing the reliability of the evaluations. These results highlight the AI’s coach capacity to deliver personalized, actionable, and empathetic coaching aligned with core behavioral science principles.

**Table 4.** Expert evaluation on simulated conversations.

	Expert 1 average rating (n=30)	Expert 2 average rating (n=30)	Interrater reliability (%) and average absolute difference in rating
Dimension (Yes=0; No=1)			
Barrier identification accuracy	0.93	0.90	80^a^
Tactic comprehensiveness	0.70	0.90	80^a^
Dimension (5-point Likert), mean (SD)			
Tactic personalization	4.38 (0.94)	4.79 (0.49)	0.78^b^
Tactic actionability	4.17 (1.10)	4.59 (0.63)	0.89^b^
Conversation empathy	4.58 (0.73)	4.76 (0.44)	0.56^b^

a Values correspond to interrater reliability.

b Values correspond to average absolute difference in rating.

#### Comparative Study

As seen in Table 5, the results of the comparative study demonstrated a preference for the behavioral science-informed workflow. The GPT-4o auto-evaluator preferred the conversations generated by the multi-agent workflow in 102 out of 153 (66.7%) cases, compared to 51 out of 153 (33.3%) cases for the single-agent base model. The blinded human expert evaluation of a subset of these conversations produced consistent results with the auto-evaluator, with both experts showing preference for the multi-agent workflow (in 66%-73% of cases) as compared with the single-agent (26%-33% of cases). To ensure a fair comparison, we report the conversation lengths, with average character counts of 3825 for the multi-agent workflow and 3904 for the base model. In addition to alternating the order presented to the auto-evaluator, the mitigation of length bias was important for ensuring that the auto-evaluator’s preferences were based on the quality of behavioral science content rather than superficial factors.

**Table 5.** Comparative evaluation on simulated conversations with behavioral science agentic workflow and base Gemini.

	No. of conversations preferred, n (%)		Conversations avg. Char. length (N=153), mean (SD)
	Autoevaluation (N=153)	Human expert review (N=30)	
Behavioral science agentic workflow	102 (66.7)	Expert 1: 22 (73.3) Expert 2: 20 (66.7)	3825 (1678)
Base Gemini	51 (33.3)	Expert 1: 8 (26.7) Expert 2: 10 (33.3)	3904 (2056)

Anecdotally, our review of the conversations revealed that the base model frequently provided more generic advice, often suggesting alternative goals or broad solutions rather than leveraging nuanced behavioral science tactics tailored to overcome the patient’s original nutrition goals. In contrast, the multi-agent workflow generated more specific and contextually relevant strategies, highlighting the benefits of structured behavioral science integration in digital coaching conversations.

## Discussion

### Overall Learnings

Our findings offer important implications for the integration of behavioral science principles into multi-agent AI coaching workflows, particularly for managing cardiometabolic conditions. The results demonstrate that our novel workflow, which utilized comprehensive barrier identification and strategy mapping, has the potential to significantly enhance the effectiveness of digital coaching interventions. The real-world user study confirmed that the workflow provides relevant, personalized support that resonates with users, fostering trust and engagement. Additionally, the introduction of structured patient simulators allowed us to systematically evaluate AI’s performance across diverse scenarios, providing a scalable method to refine and validate the system’s approach. By moving beyond surface-level motivational techniques, our approach directly targeted the root causes of nutrition-related behaviors, offering personalized and tailored coaching experiences. The strong preference for our workflow, as evidenced by both human expert evaluations and auto-evaluation, underscores the potential of structured, behaviorally informed AI coaching systems to deliver more nuanced and relevant guidance. This study sets a precedent for the development of agentic workflows that can effectively adapt to individual patient contexts, fostering sustained engagement and positive health outcomes through a deep understanding of patient-specific barriers and strategies.

### System Design Implications Beyond This Research Work

The implications of our findings extend beyond the cardiometabolic nutrition domain, suggesting that personalized AI coaching systems have the potential to play a transformative role in the future of digital health interventions. By aligning coaching systems with individual patient needs, these AI models can improve patient engagement, which is often a major barrier in digital health tools. Our results reinforce the growing importance of integrating behavioral science into AI systems to ensure that the interventions proactively help patients navigate their health goals in a structured, effective way. This also opens the door for AI to contribute more meaningfully to other areas of health management, such as physical activity management, mental health support, and preventive care.

### Limitations and Future Research Opportunities

Our study has several limitations that highlight opportunities for future research. First, the sample size for both the user research study and human expert evaluation was relatively small, which may limit the generalizability of the findings. Future research should replicate these studies with larger, more diverse populations over time to confirm the effectiveness of the multi-agent LLM workflow in broader contexts. Future research could also refine barrier identification by introducing direct questioning by the agent to the user about whether they feel equipped to overcome their barriers, rather than relying on conversational inferences. In addition, although the use of auto-evaluation enables scalable assessment, there are known biases when using LLMs as judges despite our best efforts to mitigate them [33-36]. Incorporating a diverse set of human evaluators could provide richer insights into the system’s real-world applicability and user experience. Moreover, considering feedback mechanisms and information retrieval approaches will create additional opportunities to improve the workflow. For instance, user feedback loops can enable reinforcement learning techniques to improve model performance, and retrieval-augmented generation approaches can ensure that the agent’s knowledge base is up-to-date and based on latest evidence [37-39]. This optimization research could focus on the long-term impact of AI-driven coaching, measuring (and testing strategies for improvement of) long-term adherence and durability of healthy nutrition habits.

Another layer of future research during real-world deployment could focus on the need for ongoing oversight to ensure safety, efficacy, and ethical compliance, with the goal of determining whether (or to which extent) it would be possible for the system to function with minimal supervision [36,40,41]. In this regard, studies that monitor specific use cases and potential risks, particularly the occurrence of hallucinations, and investigate mitigation strategies are warranted. Finally, real-world operational challenges related to scalability, integration into health care systems, and ensuring equitable access must be carefully evaluated to maximize the positive impact of AI-driven coaching on diverse populations [37,42].

### Conclusions

In conclusion, this study presents a novel multi-agent LLM workflow that leverages behavioral science principles to enhance digital coaching for nutrition management among individuals with cardiometabolic conditions. Our approach, validated through expert assessments, real-world user studies, and large-scale simulation-based evaluations, provides strong evidence for the effectiveness of AI-driven, personalized coaching systems that go beyond generic advice to offer tailored, actionable, and empathetic guidance. Although further research is needed to validate these findings across larger and more diverse populations, our results pave the way for the development of scalable, behaviorally informed AI systems that can support meaningful and sustained health behavior change, addressing both current limitations and future possibilities in digital health interventions.

## Supplementary material

10.2196/75421 Multimedia Appendix 1 Supplementary methods and results.

## Abbreviations

AI: artificial intelligence
BCT: Behavioral Change Taxonomy
COM-B: capability-opportunity-motivation-behavior
EAST: Easy, Attractive, Social, and Timely
LLM: large language models
