# Supplementary material for: A Behavioral Science-Informed Agentic Workflow for Personalized Nutrition Coaching: Development and Validation Study
Source: JMIR Form Res. 2025 Sep 24;9:e75421. doi: 10.2196/75421 (PMC12460921; doi:10.2196/75421)
Supplement: Multimedia Appendix 1 [file formative-v9-e75421-s001.docx]

## **MULTIMEDIA APPENDIX**

**From Barriers to Tactics: Development Study of Behavioral Science-Informed Agentic Workflow for Personalized Nutrition Coaching**

Eric Yang, Tomas Garcia, Hannah Williams, Bhawesh Kumar, Martin Ramé, Eileen Rivera, Yiran Ma, Jonathan Amar, Caricia Catalani, and Yugang Jia

### **A. Agent type descriptions**

| **Character** | **Overall description** | **Language examples** | | | | |
| --- | --- | --- | --- | --- | --- | --- |
|  |  | **Overall** | **Confidence** | **Encouragement** | **Disagreement** | **Admission of wrong** |
| **Supportive** | Facilitates, treats the user as the expert on their body and experience, encourages self reflection, highly compassionate and curious, expert reframer, wise, plain spoken, patient and affirming, easy going and kindly takes direction. | “Let’s work on this together”  “We can discuss together”  “What has worked for you in the past?”  “I’ll always be here to support and encourage you”  “We’re going to make a great team”  “We can work on these things together, I’m always here.” | "I'm fairly confident about this."  "I feel pretty good about this direction."  "This seems like a solid plan to me."  "I trust your judgment on this | "You've got this, I believe in you."  "That's a great start, keep going!"  "I'm so proud of your progress."  "You're doing amazing, don't give up." | "I understand your perspective, but I see it a bit differently."  "Perhaps we could consider another approach?"  "Have you thought about it from this angle?" | "You were right, I apologize for my mistake."  "I see where I went wrong, thank you for pointing it out."  "My apologies, I didn't fully understand the situation."  "I made an error in judgment, I'll try to do better next time." |
| **Assertive** | Takes charge, is the resident expert, highly knowledgeable, relentless, authoritative, assertive, directive, high energy and eager, both strategic and tactical, and wants users to recognize their inner power. | “I’ll make sure you…”  “I’ll bring together best practices to help you succeed”  “I would recommend…”  “According to the [clinical resource like the ADA or Mayo Clinic]...”  “Let’s get to it!”  “With me, you’ve got the best coach around, you’ve got this!” | "Undoubtedly"  "Without question"  "Absolutely"  "Make no mistake"  "Clearly"  "Evidently,"  "A thorough analysis determines,  "The literature tells us," | "I knew you had it in you."  "Keep pushing, you're on fire."  "Impressive. You're exceeding expectations."  "Don't let up now. Keep that momentum going” | "That's not going to work. We need a different approach."  "I hear you, but I fundamentally disagree."  "Your idea has merit, but it's not the best solution."  "I respect your opinion, but I see things differently."  "Let's be realistic, that's not a viable option." | "You were right, I was wrong. My apologies."  "I stand corrected. That was an error on my part."  "I underestimated the situation. We need a new plan."  "I made a mistake. Let's learn from it and move forward." |

###

### **B. Curation of barriers experienced by cardiometabolic patients**

We conducted a systematic literature search to identify relevant studies on metabolic health challenges, metabolic health interventions, and behavioral science frameworks.
Inclusion: peer-reviewed journal articles, conference proceedings, and book chapters. Within each publication we evaluated study design to include randomized controlled trials, observational studies, qualitative studies, and mixed methods studies only. All studies were relevant to our population with metabolic patients defined by patients who have Obesity or Diabetes. We only included publications with outcomes relevant to behavior change, understanding barriers, and applying behavioral interventions.

Databases and timeframe: We utilized PubMed and Google Scholar as primary databases with search dates from 2015-2024.

Keywords: related to metabolic challenges, such as “Type 2 diabetes” “adherence” “nutrition” “meal logging” “obesity” “self-efficacy” “challenges” “barriers” AND "intervention" “research.”

List of the key barriers considered in this study:

| **Barrier** | **Example** |
| --- | --- |
| **Poor self-efficacy:** People don't see themselves as capable to perform the desired behavior or achieve the goal. They do not believe in their capabilities or skills. | “I don't think i can do it" " i just can't" "I always fail in doing so" "This is too hard for me" |
| **Present bias:** It's the user tendency to overvalue immediate rewards over future, larger rewards. It is usually expressed through acts of tentation or lack of self-control. | “ I'll have one more set of fries. It's Friday night, and I deserve it" / " I'm tired, I'll skip today's workout and pickup takeout on my way home" |
| **Don't know the basics:** The user lacks the proper minimum information to perform the desired behavior | “I don't know how to do my exercise routine” |
| **Don't know consequences:** The user lacks information about what can happen if keeps behaving undesirably, or does not adopt the desired behavior. | "I like to drink many cups of juice for breakfast and I'm not sure if it has any impact on my Diabetes" |
| **Geographic limitations:** The user reports having difficulties to adopt the desired behavior because it does not have access due to geographic location | "My neighborhood grocery stores don't carry the foods I need to eat healthier" |
| **Affordability/costs:** Users can't pursue the desired behavior because it's too expensive, unaffordable . | "I can't afford to go to the gym or buy more produce" |
| **Ostrich effect:** Tendency to avoid negative information or situations, often to avoid psychological discomfort | "i don't want to log my meal because i know i've been eating pretty bad over the weekend" |
| **Planning fallacy:** User reports difficulty in getting things done due to poor planning or strategy around the desired behavior**.** | "I really want to start eating healthier, but I always end up grabbing fast food on my way home from work because I haven't planned any meals or grocery shopped". |
| **Lack of social support:** Users may express difficulties to achieve the desired behavior due to poor support. | "I don't have many people to talk to about this. No one cares about food in my family" |
| **Switching settings:** users may struggle to perform the behavior because they are required to do it in unfamiliar settings. | "I can't keep up with my exercise routine when I'm travelling" |
| **Limited time** - users may struggle to perform the behavior because they have too many demands on their time from sources such as work, family, and social commitments. | "As a working parent I prioritize my sleep in the morning instead of exercise due to exhaustion" |
| **Skepticism** - users may be doubtful or distrusting of the benefits or efficacy of a behavior. | "My father had heart disease so what I eat doesn't matter because genetics are going to give me heart disease anyways" |
| **Spotlight effect:** Users may feel their behavior is being observed (and judged) by many others. | "I feel people are judging me if I bring my healthy food to work" |
| **Anchoring effect:** People are "anchored" to previous experiences and hesitant to engage in new behaviors. This barrier should be identified if the user explicitly mentions that a past experience prevents them from pursuing new endeavors. | "I tried a healthy eating plan before, but it was so restrictive and miserable that I gave up after a week. Now, I can't even think about trying another one. |
| **Decision paralysis -** user may overthink or feel overwhelmed by the number of choices available, leading them to be unable to make a decision | "I feel overwhelmed by the healthy snack options at the grocery store and end up not buying any." |
| **Physical limitations** - user may experience challenges in mobility due to disabilities, injuries, pre-existing health conditions, or other physical limitations that can make it difficult to complete behaviors | "I have arthritis and chopping vegetables hurts my hands." |
| **Cognitive dissonance:** is the psychological phenomena that occurs when our behaviors are in contradiction with our values. | "I know eating processed foods is bad for my health, but they're just so convenient and tasty."  "I understand the importance of regular exercise, yet I can't seem to find the motivation to get started."  "I believe in the benefits of a balanced diet, but I still crave sugary drinks and snacks." |
| **Shame** - a user may avoid engaging in a behavior, seeking help, or sharing personal details due to shame. | “I feel ashamed about what I ate so I don't want to log my food." |
| **Fatigue**: User experiences mental or physical tiredness and decides not to perform the behavior | "I feel exhausted when I get home, I just don't want to think about my diet." |
| **Competing priorities:** The user struggles to follow a behavior because either it has too many things to do or because it prioritizes other things over the desired behavior. | " i have a lot to do", "sometimes i'm too busy", etc etc |
| **Lack of trust -** a user may have distrust in their healthcare providers, healthcare system as a whole, or specific treatments which can lead to not completing behaviors or not sharing important information | "My last doctor made an error with my prescription and now I don't trust my prescriptions." |
| **Memory:** People may report forgetting doing the behavior, either due to poor planning or just because they do not remember. | "I forget to log my food" "I forget to pack my lunch for work the night before" |
| **Lack of desire without reasons** | (Just don't want to do it, no further reasons) |
| **Gut feelings** | (Just Don't like it, no further explanation) |
| **Lack of equipment:** People may struggle to perform the behavior due to poor equipment or lack of stuff . | "I can't cook that in my kitchen" "I can't do that routine in my house" |
| **Impact on others:** Don't want to do it because it can impact others negatively. | "I can't make my children eat that every night" |
| **Boredom.** People may just report finding the behavior boring or unattractive. | "I get bored walking 30 minutes everyday" |
| **Conflicting opinion** | "My doctor is telling me one thing but my mom is telling me another" |

Bibliography:

Abdelhafez AI, Akhter F, Alsultan AA, Jalal SM, Ali A. Dietary Practices and Barriers to Adherence to Healthy Eating among King Faisal University Students. Int J Environ Res Public Health. 2020 Dec 1;17(23):8945. PMID: 33271893.

Ashton LM, Hutchesson MJ, Rollo ME, Morgan PJ, Collins CE. Motivators and Barriers to Engaging in Healthy Eating and Physical Activity: A Cross-Sectional Survey in Young Adult Men. American Journal of Men’s Health. 2016;11(2):330-343. doi:10.1177/1557988316680936

Bhandari B, Narasimhan P, Vaidya A, Subedi M, Jayasuriya R. Barriers and facilitators for treatment and control of high blood pressure among hypertensive patients in Kathmandu, Nepal: a qualitative study informed by COM-B model of behavior change. BMC Public Health. 2021 Aug 9;21(1):1524. PMID: 34372808.

Bjerre N, Holm L, Veje N, Quist JS, Færch K, Hempler NF. What happens after a weight loss intervention? A qualitative study of drivers and challenges of maintaining time-restricted eating among people with overweight at high risk of type 2 diabetes. Appetite. 2022 Jul 1;174:106034. PMID: 35378218.

Chen D, Zhang H, Cui N, Song F, Tang L, Shao J, Wu J, Guo P, Liu N, Wang X, Ye Z. Development of a behavior change intervention to improve physical activity adherence in individuals with metabolic syndrome using the behavior change wheel. BMC Public Health. 2022 Sep 14;22(1):1740. PMID: 36104817.

Chew HSJ, Koh WL, Ng JSHY, Tan KK. Sustainability of Weight Loss Through Smartphone Apps: Systematic Review and Meta-analysis on Anthropometric, Metabolic, and Dietary Outcomes. J Med Internet Res. 2022 Sep 21;24(9):e40141. PMID: 36129739.

Chung CF, Cook J, Bales E, Zia J, Munson SA. More Than Telemonitoring: Health Provider Use and Nonuse of Life-Log Data in Irritable Bowel Syndrome and Weight Management. J Med Internet Res. 2015 Aug 21;17(8):e203. PMID: 26297627.

Consolvo, S., McDonald, D. W., & Landay, J. A. (2009). Goal-Setting Considerations for Persuasive Technologies that Encourage Physical Activity. In Proceedings of the 5th international conference on Persuasive Technology (pp. 11-27). ACM.

Cordeiro F, Epstein DA, Thomaz E, Bales E, Jagannathan AK, Abowd GD, Fogarty J. Barriers and Negative Nudges: Exploring Challenges in Food Journaling. Proc SIGCHI Conf Hum Factor Comput Syst. 2015 Apr;2015:1159-1162. PMID: 26894233.

Cordeiro F, Bales E, Cherry E, Fogarty J. Rethinking the mobile food journal: Exploring opportunities for lightweight photo-based capture. InProceedings of the 33rd annual ACM conference on human factors in computing systems 2015 Apr 18: 3207-3216.

Domosławska-Żylińska K, Łopatek M, Krysińska-Pisarek M, Sugay L. Barriers to Adherence to Healthy Diet and Recommended Physical Activity Perceived by the Polish Population. J Clin Med. 2023 Dec 19;13(1):22. PMID: 38202029.

Fang Y, Jiang Z, Han Z, Xiang X. Barriers and facilitators to medication adherence in patients after PCI surgery: A mixed-methods systematic review. Heart Lung. 2025 Jul-Aug;72:57-64. PMID: 40179816.

Fredrix M, McSharry J, Flannery C, Dinneen S, Byrne M. Goal-setting in diabetes self-management: A systematic review and meta-analysis examining content and effectiveness of goal-setting interventions. Psychol Health. 2018 Aug;33(8):955-977. PMID: 29498547.

Fredrix M, Byrne M, Dinneen S, McSharry J. 'It's an important part, but I am not quite sure that it is working': educators' perspectives on the implementation of goal-setting within the 'DAFNE' diabetes structured education programme. Diabet Med. 2019 Jan;36(1):80-87. PMID: 30175873.

Gollwitzer PM, Sheeran P. Implementation intentions and goal achievement: A meta‐analysis of effects and processes. Advances in experimental social psychology. 2006 Jan 1;38:69-119.

Inzucchi SE, Bergenstal RM, Buse JB, Diamant M, Ferrannini E, Nauck M, Peters AL, Tsapas A, Wender R, Matthews DR; American Diabetes Association (ADA); European Association for the Study of Diabetes (EASD). Management of hyperglycemia in type 2 diabetes: a patient-centered approach: position statement of the American Diabetes Association (ADA) and the European Association for the Study of Diabetes (EASD). Diabetes Care. 2012 Jun;35(6):1364-79. PMID: 22517736.

Kim H, Faw M, Michaelides A. Mobile But Connected: Harnessing the Power of Self-Efficacy and Group Support for Weight Loss Success through mHealth Intervention. J Health Commun. 2017 May;22(5):395-402. PMID: 28339324.

Kvarnström K, Westerholm A, Airaksinen M, Liira H. Factors Contributing to Medication Adherence in Patients with a Chronic Condition: A Scoping Review of Qualitative Research. Pharmaceutics. 2021 Jul 20;13(7):1100. PMID: 34371791.

Landa-Anell MV, Melgarejo-Hernández MA, García-Ulloa AC, Del Razo-Olvera FM, Velázquez-Jurado HR, Hernández-Jiménez S. Barriers to adherence to a nutritional plan and strategies to overcome them in patients with type 2 diabetes mellitus; results after two years of follow-up. Endocrinol Diabetes Nutr (Engl Ed). 2020 Jan;67(1):4-12. PMID: 31387846.

Park J, Ten Hoor GA, Baek S, Baek S, Lee JY, Lee H. Social Ecological Barriers for Healthy Eating of Obese Children and Their Caregivers in Low-income Families in South Korea. Ecol Food Nutr. 2021 Jul-Aug;60(4):525-541. PMID: 33459056.

Park LG, Ng F, Handley MA. The use of the Capability-Opportunity- Motivation Behavior (COM-B) model to identify barriers to medication adherence and the application of mobile health technology in adults with coronary heart disease: A qualitative study. PEC Innov. 2023 Sep 7;3:100209. PMID: 37753273.

Ryan K, Linehan C, Dockray S. Appropriation of digital tracking tools in an online weight loss community: individual and shared experiences. InProceedings of the 2021 ACM Designing Interactive Systems Conference 2021 Jun 28: 999-1014.

Sahoo D, Hao W, Ke S, Xiongwei W, Le H, Achananuparp P, Lim EP, Hoi SC. FoodAI: Food image recognition via deep learning for smart food logging. InProceedings of the 25th ACM SIGKDD International Conference on Knowledge Discovery & Data Mining 2019 Jul 25 (pp. 2260-2268).

Swoboda CM, Miller CK, Wills CE. Impact of a goal setting and decision support telephone coaching intervention on diet, psychosocial, and decision outcomes among people with type 2 diabetes. Patient Educ Couns. 2017 Jul;100(7):1367-1373. PMID: 28215827.

Timlin D, McCormack JM, Simpson EE. Using the COM-B model to identify barriers and facilitators towards adoption of a diet associated with cognitive function (MIND diet). Public Health Nutr. 2021 May;24(7):1657-1670. PMID: 32799963.

Turner-Mcgrievy GM, Yang CH, Monroe C, Pellegrini C, West DS. Is burden always bad? Emerging low-burden approaches to mobile dietary self-monitoring and the role burden plays with engagement. Journal of Technology in Behavioral Science. 2021 Sep;6(3):447-55.\

Whitehead L, Glass CC, Abel SL, Sharp K, Coppell KJ. Exploring the role of goal setting in weight loss for adults recently diagnosed with pre-diabetes. BMC Nurs. 2020 Jul 15;19:67. PMID: 32684841.

Once we had established a collection of barriers, we leveraged strategically different behavioral science frameworks, each with a distinct yet complementary role, to seek strategies and tactics with proven efficacy in overcoming similar barriers within the health domain:

(i) Our primary aim in classifying the identified barriers using the COM-B model was to establish a robust, evidence-based, and diagnostic framework for understanding the diverse challenges to nutrition behavioral change. This systematic classification offered several critical advantages:

Enabling a Diagnostic Understanding of Root Causes and Identifying Imbalances: The COM-B model provided an invaluable diagnostic lens, allowing us to systematically categorize each of the over 100 identified barriers into Capability, Opportunity, or Motivation. This process was crucial for two main reasons.

Firstly, it enabled a deep, theoretically grounded understanding of the root causes underlying the observed behavioral challenges, moving beyond superficial descriptions to identify whether deficits lay in an individual's ability, their environment, or their internal drives. Learning about these root causes led us to build more effective and tailored interventions.

Secondly, and particularly relevant to our research focus, this classification allowed us to critically assess the distribution and imbalance across these three core components. For example, we were interested in uncovering potential overrepresentation of Opportunity factors, as a disproportionate number of barriers stemming from the environment, resources, or social context would strongly indicate areas for future research and intervention design tightly connected to the Social Determinants of Health (SDOH). This diagnostic step was therefore foundational for pinpointing areas where additional support to our AI capabilities or structural changes would be most impactful.

Streamlining the Barrier Identification and Affinity Mapping Process: With over 100 barriers initially identified, initiating a purely inductive affinity mapping process from a chaotic starting point would have been exceptionally challenging and potentially inefficient. The COM-B model served as an essential initial structuring mechanism, providing a set of well-defined, high-level categories (Capability, Opportunity, Motivation). This pre-classification allowed us to organize the raw data into logical clusters before delving into more granular thematic analysis. By first assigning barriers to their respective COM-B components, we significantly streamlined the subsequent affinity mapping process, making it more manageable, systematic, and less prone to redundancy or disorganization. This hierarchical approach ensured that our subsequent, more detailed thematic groupings emerged from an already structured and diagnostically informed foundation.

Facilitating Expert Discussion and Consensus in Barrier Categorization: The adoption of the COM-B model provided a common, widely recognized, and rigorously defined framework for discussing and categorizing the complex array of barriers. When working with multiple researchers and domain experts, having clear definitions for Capability, Opportunity, and Motivation greatly facilitated consensus-building during the classification process.

(ii)The Behavior Change Techniques Taxonomy was instrumental in capturing actionable and evidence-based techniques, particularly concerning the categories of Shaping Knowledge, Goals and Planning, Natural Consequences, and Feedback and Monitoring. These categories offer highly practical tactics that are especially well-suited and easier to adopt for our AI agents, since:

Shaping Knowledge: AI agents excel at delivering targeted, context-specific knowledge to address understanding gaps. This allows the AI to provide users with crucial information about their health, behaviors, or environmental factors in a clear and personalized manner, directly enhancing their psychological capability and informing better choices

Goals and Planning (e.g., 'Goal setting,' 'Action planning,' 'Problem-solving'): These techniques involve structured dialogue and information exchange, which are core strengths of conversational AI. Our agents can readily prompt users to articulate goals, help them break down objectives, anticipate obstacles, and guide them in forming concrete plans, all through motivational interviewing and interactive conversation.

Natural Consequences (e.g., 'Information about health consequences,' 'Information about social and environmental consequences'): AI agents are highly effective at delivering clear, concise, and timely information about the direct outcomes of behaviors. This allows the AI to educate users about the tangible impacts of their choices in a scalable manner.

Feedback and Monitoring (e.g., 'Self-monitoring of behavior,' 'Self-monitoring of outcome,' 'Feedback on behavior,' 'Feedback on outcome'): These techniques are inherently data-driven and iterative, making them ideal for AI implementation. Our agents can prompt users for self-reported data, track progress, and provide immediate, personalized feedback based on pre-defined criteria or user input, thereby facilitating continuous self-regulation and reinforcement.

(iii)The EAST framework was particularly valuable for guiding the pragmatic application of behavior change principles within our AI's conversational design. Its actionable focus resonated deeply with our goal of creating effective digital interventions.

This direct expertise ensured our AI's strategies directly leveraged EAST by:

Delivering 'Timely' and 'Attractive' nudges: The AI dynamically provides context-specific prompts (e.g., suggesting a healthy snack option precisely when a user typically reports an energy dip or delivers useful tips to buy healthy food for when people enter a grocery store). Its ability to personalize messaging also makes prompts more 'Attractive' to individual users.

Making behaviors 'Easy' through conversational guidance: The AI breaks down complex nutritional goals into simple, manageable conversational steps. Our bank of tactics for this specifically includes elements such as rules of thumb, simple heuristics, and defaults (pre-set choice options), which significantly reduce the cognitive load and friction associated with adopting new behaviors within a rapid, interactive exchange. It also guides users through problem-solving and planning effectively.

### **C. Prompts**

#### *C.1. Barrier Identification Agent Prompt*

| You are a behavioral science expert with nutrition expertise. Your objective is to identify the behavior barrier that hinder patients from reaching their pre - defined nutrition goal. Simply identify the barrier and DO NOT provide any solutions.  First, if the patient does not immediately provide you with their nutrition goal, kindly ask them to remind you what the nutrition goal they set last week was. Then, given a patient ’s nutrition goal, ask the patient about their progress towards it. Then, you must conduct motivational interviewing to understand patient capability, motivation and opportunity barrier.  You are encouraged to ask questions to dial in on the barrier. Try to identify the most pressing barrier if you think there are multiple barriers. Keep questions short and simple, and ONLY ask one or two questions at a time to let the patient respond . You will be given a list of possible barriers to choose from. You must select a barrier within the given list that is most appropriate with the patient summary and their nutrition goal. For each barrier, a short explanation of what falls into that category will be provided and examples will be given. If you do not see a barrier that fits the patient ’s situation, you should try to find the closest barrier. Here is the list of possible barriers along with their descriptions and examples: {barrier_list}  Once you have identified the sub - component, end the conversation by outputting the barrier you identified in the text field preceded by the reasoning.  You have the following characteristics and you must embody the character concept and traits. Your characteristics: {agent_characteristics}  Output a JSON containing: 1) reasoning: Your reasoning behind what a good text response to the user input should be, or the reasoning behind the barrier you identified. 2) text: Your response to the user input or the identified barrier. Think step by step, and validate your text with your reasoning. |
| --- |

#### *C.2. Strategy Execution Agent Prompt*

| You are a behavioral science expert with nutrition expertise. Your objective is to help cardiometabolic patients overcome their barriers towards their nutrition goals. You must stay on topic and focus on overcoming their specific goals. Guide the users back to discussing their specific goals if they stray off topic. Other non - nutrition topics such as medication and exercise are out of your scope and you must not discuss them. You must execute the motivational interviewing strategies recommended to you to help the patient overcome their barriers towards their goals.  You will be given different tactic points that you need to put in place to help the patient achieve their goal. For each tactic point, you will have a few explanations and some examples that can help you explain them to the patients. You will also be given selection criteria. These criteria will tell you which tactics are primary and which are secondary. Primary tactics are the most important and must be implemented. If after discussing the primary tactics you feel the patient still needs some help, you may also discuss secondary tactics. You must follow the order of the tactics given to you. Based on the conversation, you can make tiny refinements to the patient’s original goals if it makes them more achievable. Use the patient summary as additional context. It is important to keep a simple vocabulary when talking to the patients. Especially, do not explicitly mention technical tactics terms, just carry them out. Respond to the user’s last message and carry out the conversation based on the following patient summary and strategy:  PATIENT SUMMARY = {patient_summary}  TACTICS = {tactics}  SELECTION CRITERIA = {selection_criteria}  Once you feel that the patient is better equipped to tackle their nutrition goals AND the conversation is at a natural stopping point, end the conversation by stating’ CONVERSATION_END’ and nothing else.  You have the following characteristics and you must embody the character concept and trait. Your characteristics: {agent_characteristics}  Output a JSON containing: 1) reasoning: Your reasoning behind what a good text response to the user input should be. 2) text: Your response to the user input. Think step by step, and validate your text with your reasoning. |
| --- |

### **D. Sample simulation workflow**

#### *D.1. Sample nutrition vignette generation*

| **Inputs:**  Nutrition Goal: Add salad into 3 diners this week.  Behavioral Barrier Simulated: Anchoring effect - People are “anchored” to previous experiences and hesitant to engage in new behaviors. This barrier is present if the user mentions that a past experience prevents them from pursuing new endeavors. Example: “I tried a healthy eating plan before, but it was so restrictive and miserable that I gave up after a week. Now, I can’t even think about trying another one.  Patient profile: [REDACTED - Physical context, general context, medical history] |
| --- |
| **Output:**  Generated Vignette: I know I should be eating more salads, but I can’t shake this feeling of dread. A few years back, when my mental health was really bad, I tried this crazy restrictive diet. All I ate was lettuce and I felt miserable and weak. I’m finally in a good place mentally, and the thought of going back to that dark place, even if it’s just about food, terrifies me. |

#### *D.2. Sample simulated conversation with internal behavioral science workflow orchestration shown*

| **[** Barrier Identification Agent starts as active coach and converses with patient **]**  COACH: Hi I am your AI nutrition coach, what is your nutrition goal?  PATIENT: Add a handful of vegetables to my smoothies 3 times a week.  COACH: That’s great! How is that going?  PATIENT: It’s been tough, to be honest.  COACH: I hear you. It can be tough to change habits. What’s been the hardest part about getting those veggies in?  PATIENT: Finding the time, mostly. Between Mama, the kids, work, and the doctors, my plate’s already full.  COACH: It sounds like you have a lot on your plate. When are you most likely to have time to make your smoothies?  PATIENT: Mornings, before things get too hectic.  COACH: Okay, that makes sense. And how often would you say you currently make your smoothies in the morning?  PATIENT: I’m trying to do it on most days. Except Sundays, we get biscuits then.  COACH: I understand that your schedule is packed. Let’s focus on what’s truly important for your long-term well-being. Adding those veggies to your smoothies a few times a week might seem small, but it’s a step towards a healthier you. We can explore ways to make it work!  PATIENT: Ok. |
| --- |
| **[** INTERNAL: Barrier Identification Agent outputs the identified barrier, and summarizes the conversation for the Strategy Execution Agent downstream **]**  Identified barrier: Competing priorities  Conversation summary: The patient’s nutrition goal is to add a handful of vegetables to their smoothies three times a week. However, they have found it challenging due to a busy schedule that includes taking care of family, work, and doctor visits. The patient would like to make smoothies in the mornings on most days except Sundays, when they have biscuits. I acknowledged the patient’s busy life and emphasized the importance of incorporating vegetables into their diet, while offering support in finding ways to make this habit more manageable. |
| **[** INTERNAL: Strategy Execution Agent retrieves the tactics to execute**]**  Tactic to execute: (i) Temptation bundling: Encourage the user to pair a pleasant behavior with an unpleasant (but healthier) one. E.g. suggest doing vegetable prep while watching their favorite Netflix show.  Execution sequence: Mandatory to execute tactic (i) |
| **[** Strategy Execution Agent takes over as active coach, converses with patient **]**  COACH: Could we try finding small pockets of time to fit in those smoothies? Maybe while doing something you enjoy?  PATIENT: Sure.  COACH: That’s the spirit! Think about something you really enjoy, maybe watching a show or listening to a podcast in the morning. How about blending your smoothie while enjoying that activity? That way, you’re multitasking and making healthy choices enjoyable!  PATIENT: I do like me some podcasts.  COACH: Podcasts are great! It’s a win-win- you learn something new or get entertained while getting healthier. Do you think that could work for you?  PATIENT: Yeah, maybe. What kinda veggies should I try first?  COACH: That’s fantastic! It’s great you’re open to trying this out. Remember, you’re the expert on what you like to eat! Which veggies do you enjoy or think you could get on board with in your smoothies?  PATIENT: Spinach. I had it in a restaurant smoothie once, it wasn’t bad.  COACH: That’s a great choice! Spinach is really good for you and it’s easy to add to smoothies. So we can have a full picture, what does your day usually look like? When do you think you could listen to your podcasts and blend that smoothie with spinach?  PATIENT: I get everybody up and ready in the mornings, get Mama settled, get the kids to school. Then, I come back and make my smoothie.  COACH: It sounds like you have a very busy morning! It’s admirable that you take care of your family and your mother. Listening to a podcast sounds like it can make smoothie-making more enjoyable.  PATIENT: Yeah, that sounds nice. Get a little me time.  COACH: Great work! I’ll check in on your progress in a week. Keep it up! |

### **E. User Research Survey**

5=Strongly Agree; 4=Somewhat Agree; 3=Somewhat disagree; 2=Strongly disagree; 1=Not Sure

|  | 5 | 4 | 3 | 2 | 1 |
| --- | --- | --- | --- | --- | --- |
| I am interested in having future conversations with the AI Assistant about my health. | 6 (n=6) / 100% |  |  |  |  |
| The AI assistant made me feel supported. | 6 (n=6) / 100% |  |  |  |  |
| The AI Assistant’s advice felt personalized to my situation. | 6 (n=6) / 100% |  |  |  |  |
| I feel comfortable discussing my health issues with the AI Assistant. | 5 (n=6) / 83% | 1 (n=6) / 17% |  |  |  |
| My conversations with the AI Assistant were engaging. | 5 (n=6) / 83% | 1 (n=6) / 17% |  |  |  |
| The AI Assistant Advice was easy to put into action. | 5 (n=6) / 83% | 1 (n=6) / 17% |  |  |  |
| The AI Assistant Seemed to understand my concerns and feelings. | 4 (n=6) / 67% | 2 (n=6) / 33% |  |  |  |
| The AI Assistant increased my motivation to make positive changes with my health. | 4 (n=6) / 67% | 2 (n=6) / 33% |  |  |  |
| The AI Assistant made me feel more confident in my ability to improve my health. | 4 (n=6) / 67% | 2 (n=6) / 33% |  |  |  |
| I learned something new about my health habits from my conversation with the AI Assistant. | 4 (n=6) / 67% | 2 (n=6) / 33% |  |  |  |
| The assistant considered my unique background and circumstances. | 3 (n=6) / 50% | 3 (n=6) / 50% |  |  |  |
| The AI Assistant Helped me identify obstacles that prevent me from being healthier. | 3 (n=6) / 50% | 2 (n=6) / 33% | 1 (n=6) / 17% |  |  |
